# Baicalein alleviates tubular-interstitial nephritis *in vivo* and *in vitro* by down-regulating NF-κB and MAPK pathways

**DOI:** 10.1590/1414-431X20187476

**Published:** 2018-08-06

**Authors:** Yan Chen, Yu Zheng, Zhihong Zhou, Jinjun Wang

**Affiliations:** 1 Department of Nephrology, The Second Affiliated Hospital and Yuying Children’s Hospital of Wenzhou Medical University, Wenzhou, China; 2 Department of Transplantation, The First Affiliated Hospital of Wenzhou Medical University, Wenzhou, China

**Keywords:** Tubular-interstitial nephritis, Baicalein, Lipopolysaccharide, Anti-inflammatory effects, NF-κB pathway, MAPK pathway

## Abstract

Tubular-interstitial nephritis (TIN) is characterized by tubular cell damage and inflammatory lesions of kidneys. Baicalein (BAI) is a flavonoid compound found in the roots of *Scutellaria baicalensis Georgi*. The present study was undertaken to explore the anti-inflammatory and anti-oxidative effects of BAI on TIN patients and a lipopolysaccharide (LPS)-induced TIN cell model. The expression levels of interleukin-6 (IL-6), IL-10, and tumor necrosis factor α in serum samples of TIN patients and culture supernatants of renal proximal tubular epithelial cells (RPTECs) were evaluated using enzyme-linked immunosorbent assay. Creatinine clearance was calculated using the Cockcroft-Gault equation. Activities of malondialdehyde, superoxide dismutase, and glutathione peroxidase were also determined. Viability and apoptosis of RPTECs were measured using MTT assay and Guava Nexin assay, respectively. qRT-PCR was performed to determine the expressions of Bax, Bcl-2, nuclear factor kappa B (IκBα), and p65. Protein levels of Bax, Bcl-2, IκBα, p65, c-Jun N-terminal kinase, extracellular regulated protein kinases, and p38 were analyzed using western blotting. We found that BAI reduced inflammation and oxidative stress *in vivo* and *in vitro*. Moreover, BAI alleviated the LPS-induced RPTECs viability inhibition and apoptosis enhancement, as well as nuclear factor kappa B (NF-κB), and mitogen-activated protein kinase (MAPK) activation. Phorbol ester, an activator of NF-κB, attenuated the effects of BAI on LPS-induced inflammatory cytokine expressions in RPTECs. In conclusion, BAI had anti-inflammatory and anti-oxidative effects on TIN patients and LPS-induced RPTECs by down-regulating NF-κB and MAPK pathways.

## Introduction

Tubular-interstitial nephritis (TIN) is characterized by tubular cell damage and inflammatory lesions of kidneys (1). Epidemiology research indicates that TIN is one of the most common diseases of kidneys (2). Common clinical symptoms of TIN include edema, hypertension, and proteinuria (3). At present, only symptomatic treatment is available for TIN due to its unclear pathogenesis (4). More experimental and clinical research is needed to explore the pathogenesis of TIN. In addition, searching for novel and more effective therapies for TIN is also urgently needed.

Lipopolysaccharide (LPS) is a component of the outer membrane of gram-negative bacteria (5). A study demonstrated that LPS could activate inflammatory response and oxidative stress *in vitro* and *in vivo* (6). Cell-based TIN models induced by LPS treatment have been widely used for testing new anti-inflammatory medicines for TIN (7).

Baicalein (BAI) is a flavonoid compound found in the roots of *Scutellaria baicalensis Georgi* (8). Previous studies have reported that BAI exerts many pharmacological effects including antimicrobial, anti-inflammatory, antihypertensive, antispasmodic, and anticancer (9–12). For anti-inflammatory effects, Patwardhan et al. demonstrated that BAI could suppress mitogen-induced T cell activation, proliferation, and cytokines secretion by down-regulating nuclear factor kappa B (NF-κB) transactivation (13). Fan et al. (14) proved that BAI exhibited anti-inflammatory activity in LPS-stimulated RAW264.7 macrophages through estrogen receptor- and NF-κB-dependent pathways. Furthermore, Cai et al. (15) reported that BAI protected tissues against periodontitis in rats by suppressing expressions of cyclo-oxygenase 2 and inducible nitric oxide synthase. He et al. (16) indicated that BAI attenuated inflammatory responses in LPS-induced mastitis in mice by inhibiting NF-κB and mitogen-activated protein kinase (MAPK) pathways. The above findings suggest that BAI has wide anti-inflammatory effects and imply that BAI can be a potential therapeutic medicine for inhibiting inflammatory injury in TIN.

Therefore, this study was aimed to explore the anti-inflammatory and anti-oxidative effects of BAI on TIN patients and primary human RPTECs induced by LPS. The possible underlying mechanisms involved in the NF-κB and MAPK pathways were also investigated.

## Material and Methods

### Sample collection

Thirty patients (12 males and 18 females, 18 to 55 years old) diagnosed with TIN and fifteen healthy people (7 males and 8 females, 21 to 46 years old) were enrolled in this study between 2012 and 2014 at The Second Affiliated Hospital and Yuying Children's Hospital of Wenzhou Medical University. The present study was approved by the Ethics Committee of the institution (approval number: IACUC-14-013). Written informed consent was obtained from all patients and healthy people.

### Experimental treatment

Patients were randomly allocated into either the TIN group or TIN+BAI group with fifteen patients in each group. Fifteen healthy people acted as the control group. Patients in the TIN group did not receive any treatment during the experiment and patients in TIN+BAI group received BAI capsules (80 mg, Xiuzheng Pharmaceutical Group, China) with 1.2 g each, 3 times per day orally for 1 month. Immediately after the experiment, serum samples of all patients and healthy people were collected to measure the levels of interleukin (IL)-6, IL-10, and tumor necrosis factor α (TNF-α), activities of malondialdehyde (MDA), superoxide dismutase (SOD), and glutathione peroxidase (GSH-PX), as well as creatinine clearance (CCr).

### Cell culture and treatment

Primary human RPTECs were purchased from FMG-Bio (Sciencell, Shanghai, China), cultured in REBM/REGM bullet kit (Biowhittaker Inc., USA) following the manufacturer's instruction and confirmed by staining using γ-glutamyl transferase (Thermo Fisher Scientific, USA), a selective marker residing in the brush border of human RPTECs (17). LPS (Sigma-Aldrich, USA) was diluted in endotoxin-free sterile phosphate-buffered saline (PBS, Sigma-Aldrich) according to the manufacturer's protocol. RPTECs were treated with 1 μg/mL LPS for 24 h to stimulate inflammatory injury in this research (18). For BAI treatment, 100 μM BAI was added into the culture medium simultaneously with LPS exposure. Phorbol ester (PMA, Sigma-Aldrich) was used as the activator of NF-κB pathway.

### Cytokine expression assay

The levels of cytokines (IL-6, IL-10, and TNF-α) in serum and in culture supernatant of RPTECs after relevant treatment were measured using enzyme-linked immunosorbent assay (ELISA) kits (R&D Systems, USA) according to the manufacturer's instructions. RPTECs were plated into 24-well plates (Corning Inc., USA) and treated with 1 μg/mL LPS and/or 100 μM BAI for 24 h. After incubation, 100 μL aliquot of culture medium supernatant from each well was collected with Transferpettor (Thermo Fisher Scientific).

### CCr analysis

CCr (mL·s^-1^·(m^2^)^-1^) was calculated using the Cockcroft-Gault equation (140 – age in years) × body weight (kg)/72/serum creatinine (Cr) (mg/dL), and × 0.85 if female) according to a previous study (19).

### MDA, SOD, and GSH-PX analysis

The activity of MDA in serum and RPTECs after treatment was measured using OxiSelect^TM^ TBARS Assay kit (Cell Biolabs, USA) according to the manufacturer's instruction. SOD activity in serum and RPTECs was evaluated by the adrenaline method according to Misra and Fridovich (20). GSH-PX activity in serum and RPTECs was measured by the method with Ellman's reagent of Sedlak and Lindsay (21), as modified by Little and O'Brien (22).

### Cell viability assay

MTT assay was performed to measure cell viability. Briefly, human RPTECs were seeded into 96-well plates (Corning Incorporated, USA) with 10^4^ cells/well and treated with 1 μg/mL LPS and/or 100 μM BAI for 24 h. Then, 10 μL MTT solution (5 mg/mL in PBS) was added into the each well of the plate followed by incubation for 4 h at 37°C. After that, the supernatant of each well was removed and 150 μL dimethyl sulfoxide (DMSO, Beyotime, China) was added into the each well. Samples were agitated on a shaker for 15 min, and the absorbance of each well at 570 nm was recorded using a microplate reader (Dynatech Laboratories, USA). Cell viability (%) was determined as percent viability of the control group, which was taken as 100%.

### Cell apoptosis assay

Apoptosis of RPTECs was determined using Guava Nexin assay after LPS and/or BAI treatment. Briefly, RPTECs were seeded into 24-well plates with 3 × 10^4^ cells per well and treated with 1 μg/mL LPS and/or 100 μM BAI for 24 h. Then, cells in each group were harvested and stained with 100 μL kit solution for 25 min at 37°C in the dark. After that, Guava easyCyte 8HT (Millipore, USA) was performed to record cell apoptosis, and data was analyzed using FCS Express software (De Novo Software, USA).

### Western blotting

Briefly, the total proteins in RPTECs were isolated using RIPA lysis and extraction buffer (Thermo Fisher Scientific) and quantified using Pierce^TM^ Rapid Gold BCA Protein Assay Kit (Thermo Fisher Scientific). Then, equal concentrations of protein samples were separated by electrophoresis on sodium dodecyl sulfate (SDS)-polyacrylamide gels and transferred onto polyvinylidene difluoride (PVDF) membranes (Millipore) with a semidry transfer system (Bio-Rad Laboratories, USA). After that, the membranes were blocked with 5% bovine serum albumin (BSA, Sigma-Aldrich) for 1 h at room temperature and incubated with primary antibodies overnight at 4°C. All primary antibodies were prepared in 1% BSA solution with a dilution of 1:1000. After hybridization with primary antibodies, the membranes were washed three times with Tris-buffered saline and Tween (TBST, Sigma-Aldrich) for 15 min and incubated with horseradish peroxidase-conjugated secondary antibodies (ab6721, ab6788, Abcam Biotechnology, USA) for 1 h at room temperature. After addition of western blotting Luminol reagents (Santa Cruz Biotechnology, USA), signals of proteins were recorded using Bio-Rad ChemiDoc^TM^ XRS system (Bio-Rad Laboratories).

### Quantitative reverse transcriptase PCR (qRT-PCR)

The total RNA in RPTECs after relevant treatment was isolated using RNeasy mini kit (Qiagen, USA) according to the manufacturer's instructions. cDNA was reversely transcribed using High Capacity cDNA Reverse Transcription kit (Applied Biosystems, USA). To detect the expressions of Bax, Bcl-2, IκBα, and p65 in RPTECs, the Power SYBR-Green Master Mix Assay (Applied Biosystems) was used following the manufacturer's protocol. The conditions for the PCR program were set at 95°C for 15 s, 60°C for 30 s, and 72°C for 45 s for 50 cycles). The data from the qRT-PCR were analyzed by CFX manager software (Bio-Rad Laboratories). The expression of GAPDH acted as endogenous control in the respective samples.

### Statistical analysis

All experiments in this research were performed in triplicate and repeated at least three times. The statistical analysis was performed using SPSS software (SPSS Inc., USA). The data of multiple experiments are reported as means±SE. Statistical comparisons between two groups were performed using Student's *t*-test. One-way ANOVA followed by Tukey's multiple comparison test was used for comparisons involving three or more groups. The observations were considered significant with a P value of <0.05.

## Results

### BAI reduced inflammatory injury and oxidative stress of TIN patients

**Figure 1.**
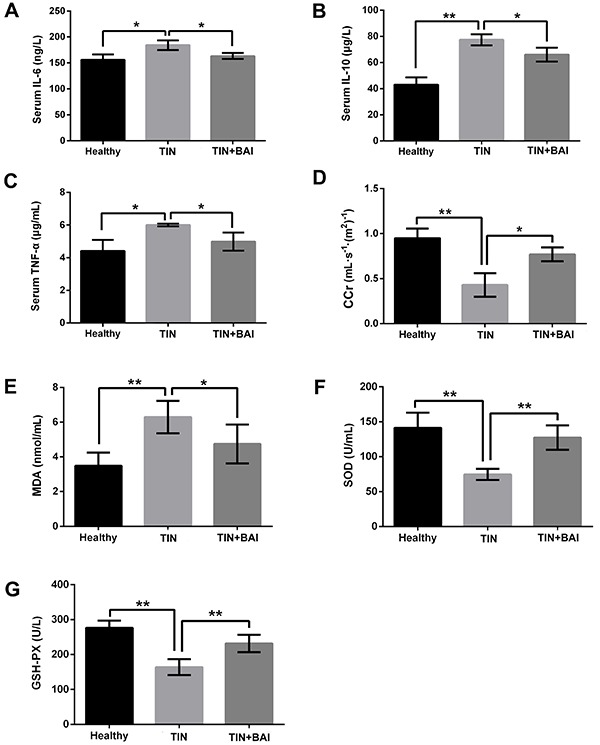
*A*–*C*, BAI treatment reduced the levels of IL-6, IL-10, and TNF-α in serum of TIN patients. *D*, BAI treatment enhanced the patient's CCr. *E*–*G*, BAI treatment reduced the activity of MDA and enhanced the activities of SOD and GSH-PX in serum of TIN patients. BAI: baicalein; TIN: tubular-interstitial nephritis; IL-6: interleukin-6; IL-10: interleukin-10; TNF-α: tumor necrosis factor-α; CCr: creatinine clearance; MDA: malondialdehyde; SOD: superoxide dismutase; GSH-PX: glutathione peroxidase. Data are reported as means±SE. *P<0.05, **P<0.01 (ANOVA).

Figure 1A and C shows that the levels of IL-6, IL-10, and TNF-α in serum samples of TIN patients with BAI treatment were significantly decreased (P<0.05). Moreover, Figure 1D shows that BAI treatment remarkably enhanced CCr level (P<0.05). The activity of MDA in serum was significantly decreased and the activities of SOD and GSH-PX in serum were increased after BAI treatment (Figure 1E–G, P<0.05 or P<0.01). These results suggested that administration of BAI significantly decreased the inflammatory injury of TIN patients.

### BAI reduced LPS-induced inflammatory response and oxidative stress in RPTECs

**Figure 2.**
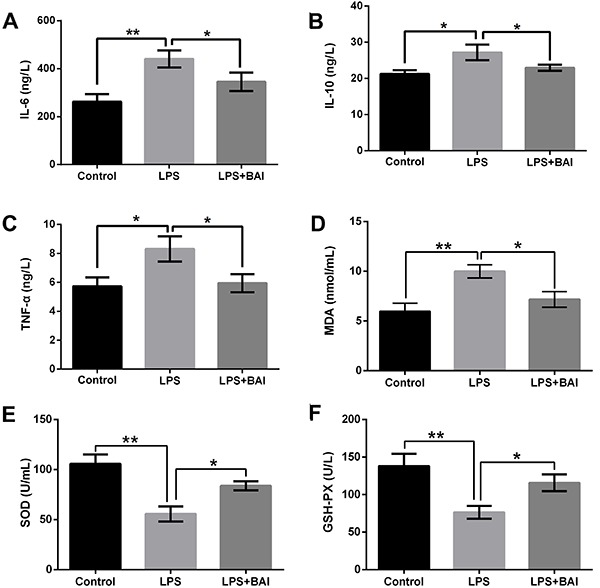
Effect of BAI in LPS-induced RPTECs in *A*, IL-6 expression; *B*, IL-10 expression; *C*, TNF-α expression; *D*, MDA activity; *E*, SOD activity; *F*, GSH-PX activity. BAI: baicalein; LPS: lipopolysaccharide; RPTECs: renal proximal tubular epithelial cells; IL-6: interleukin-6; IL-10: interleukin-10; TNF-α: tumor necrosis factor-α; MDA: malondialdehyde; SOD: superoxide dismutase; GSH-PX: glutathione peroxidase. Data are reported as means±SE. *P<0.05; **P<0.01 (ANOVA).

As shown in Figure 2A–C, LPS treatment dramatically increased the expression levels of IL-6, IL-10, and TNF-α in culture supernatant of RPTECs (P<0.05 or P<0.01). BAI co-treatment significantly alleviated the LPS-induced increases of IL-6, IL-10, and TNF-α (P<0.05). In addition, the results of Figure 2D–F display that LPS treatment significantly enhanced the activity of MDA and reduced the activities of SOD and GSH-PX in RPTECs (P<0.01). BAI co-treatment alleviated the LPS-induced MDA increase, as well as SOD and GSH-PX decreases in RPTECs (P<0.05). These findings indicated that BAI attenuated LPS-induced inflammation and oxidative stress in RPTECs.

### BAI alleviated LPS-induced RPTECs viability inhibition and apoptosis enhancement

**Figure 3.**
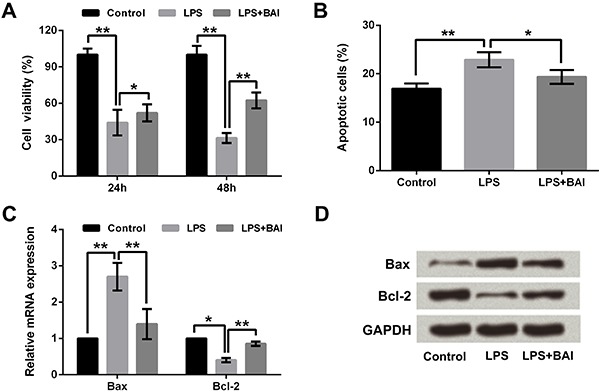
Effect of BAI on LPS-induced RPTECs. *A*, viability; *B*; apoptosis; *C*, mRNA expression of Bax and Bcl-2. *D*, Bax and Bcl-2 results by western blotting. BAI: baicalein; LPS: lipopolysaccharide; RPTECs: renal proximal tubular epithelial cells. Data are reported as means±SE. *P<0.05; **P<0.01 (ANOVA).

Viability and apoptosis of RPTECs after LPS and/or BAI treatment were analyzed using MTT assay and Guava Nexin assay, respectively. Figure 3A displays that LPS treatment significantly inhibited the viability of RPTECs in a time-dependent manner (P<0.01) and BAI co-treatment remarkably alleviated the LPS-induced RPTECs viability inhibition (P<0.05 or P<0.01). Figure 3B shows that BAI co-treatment noticeably attenuated the LPS-induced RPTECs apoptosis (P<0.05). In addition, qRT-PCR showed that, compared to LPS alone, BAI co-treatment significantly decreased the mRNA expression of Bax (P<0.01) and increased the mRNA expression of Bcl-2 in RPTECs (Figure 3C, P<0.01). Similar results were found in western blotting, which presented that BAI co-treatment attenuated the LPS-induced Bax protein level increase and Bcl-2 level decrease (Figure 3D). These findings indicated that BAI alleviated the LPS-induced RPTECs viability inhibition and apoptosis enhancement.

### BAI inhibited LPS-induced NF-κB and MAPK activation in RPTECs

**Figure 4.**
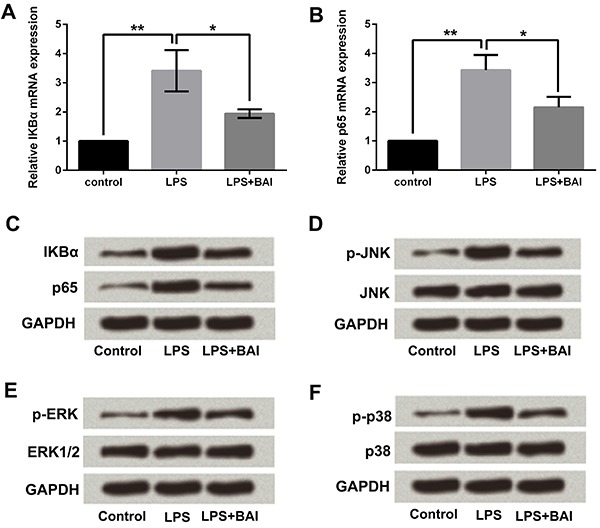
*A* and *B*, Effect of BAI on LPS-induced mRNA expression of IκBα and p65 increase in RPTECs. *C*, Representative western blotting images of protein expressions of IκBα and p65; *D*–*F*, protein expressions of p-JNK, p-ERK, and p-38. BAI: baicalein; LPS: lipopolysaccharide; RPTECs: renal proximal tubular epithelial cells; NF-κB: nuclear factor kappa B; MAPK: mitogen-activated protein kinase; IκBα: inhibitor of NF-κB; JNK: c-Jun N-terminal kinase; ERK: extracellular regulated protein kinases. Data are reported as means±SE. *P<0.05; **P<0.01 (ANOVA).

The mRNA and protein levels of IκBα and p65 in RPTECs after LPS and/or BAI treatment were analyzed by qRT-PCR and western blotting, respectively. Figure 4A and B shows that LPS incubation enhanced the mRNA levels of IκBα and p65 in RPTECs (P<0.01) and BAI co-treatment significantly alleviated the LPS-induced IκBα and p65 increases in RPTECs (P<0.05). Similar results were found in western blotting, which displayed that the protein levels of IκBα and p65 in RPTECs were also enhanced after single LPS incubation and reduced after LPS+BAI treatment (Figure 4C). Protein levels of the MAPK pathway were analyzed using western blotting. Figure 4D-F present that LPS incubation increased the protein levels of p-JNK, p-ERK, and p-p38 in RPTECs, whereas BAI co-treatment attenuated the effects of LPS on p-JNK, p-ERK1/2, and p-p38 in RPTECs. These findings suggested that BAI inhibited the LPS-induced NF-κB and MAPK pathways activation in RPTECs.

### BAI reduced LPS-induced inflammatory cytokine expressions in RPTECs by down-regulating NF-κB pathway

**Figure 5.**
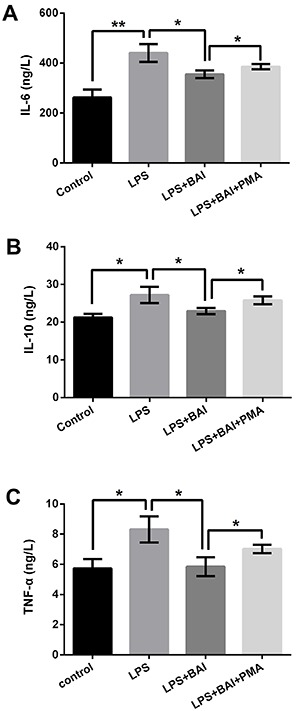
*A*, PMA alleviated the effects of BAI on LPS-induced IL-6 expression; *B*, IL-10 expression; and *C*, TNF-α expression in RPTECs. BAI: baicalein; LPS: lipopolysaccharide; RPTECs: renal proximal tubular epithelial cells; NF-κB: nuclear factor kappa B; PMA: phorbol ester; IL-6: interleukin-6; IL-10: interleukin-10; TNF-α: tumor necrosis factor-α. Data are reported as means±SE. *P<0.05; **P<0.01 (ANOVA).

To further confirm whether BAI reduces LPS-induced inflammatory cytokines in RPTECs by regulating NF-κB pathway, we administered the activator of NF-κB pathway PMA in our experiments (23). Then, the expression levels of IL-6, IL-10, and TNF-α were tested by ELISA. Results in Figure 5A–C show that the expressions of IL-6, IL-10, and TNF-α were increased in LPS+BAI+PMA treatment group compared to LPS+BAI treatment group (P<0.05). These findings suggested that incubation of PMA significantly attenuated the effects of BAI on LPS-induced inflammatory cytokine expressions in RPTECs.

## Discussion

Inflammatory processes and oxidative stress are both associated with many diseases, including TIN (24–27). Chinese herbs have gained attention worldwide in the treatment of multiple diseases, including TIN (28). In this research, we found that BAI, a flavonoid compound isolated from the roots of *Scutellaria baicalensis*, significantly reduced the inflammatory injury and oxidative stress of TIN patients, as well as LPS-induced RPTECs. Further results showed that BAI remarkably alleviated the LPS-induced RPTECs viability inhibition and apoptosis enhancement, as well as NF-κB and MAPK activation. In addition, PMA attenuated the effects of BAI on LPS-induced inflammatory cytokine expressions, which implied that BAI reduced the LPS-induced RPTECs inflammatory injury and oxidative stress at least in part by down-regulating the NF-κB pathway.

BAI is a promising anti-inflammatory medicine due to its wide anti-inflammatory effects on multiple diseases (24,26). Many previous studies demonstrated the anti-inflammatory effects of BAI (13–16). Oxidative stress is often enhanced in patients with TIN, which promotes the expressions of inflammatory cytokines, such as IL-6, IL-10, and TNF-α (29). As an important indicator of renal function, CCr is often decreased in patients with TIN (30). In this research, we found that the expression levels of IL-6, IL-10, and TNF-α were increased in serum samples of TIN patients and CCr was decreased. Moreover, the activity of MDA was increased and the activities of SOD and GSH-PX were decreased in serum samples of TIN patients. BAI treatment significantly reduced the expression levels of IL-6, IL-10, and TNF-α in serum samples, enhanced the patient's CCr, and alleviated oxidative stress. Results in RPTECs showed that BAI treatment remarkably alleviated the LPS-induced IL-6, IL-10, and TNF-α expression increases, MDA activity increase, and SOD and GSH-PX activities decreases in RPTECs. These findings suggested that BAI exerted anti-inflammatory and anti-oxidative effects on TIN patients and the LPS-induced TIN cell model.

Excessive oxidative stress can promote the expression of inflammatory cytokines (31). Superabundant inflammatory cytokines production can damage mitochondrial function and then induce cell apoptosis (32). In this research, LPS treatment inhibited RPTECs cell viability and induced cell apoptosis. The expression of Bax was increased and the expression of Bcl-2 was decreased in RPTECs after LPS treatment. BAI treatment effectively alleviated the LPS-induced cell viability inhibition and cell apoptosis enhancement. These results suggested that BAI also exerted anti-injury effects on the LPS-induced TIN cell model.

Previous studies demonstrated that BAI exerted anti-inflammatory activity by suppressing NF-κB and MAPK pathways (13,16). NF-κB and MAPK signaling pathways play critical regulatory roles in multiple cell functions including cell proliferation, apoptosis, autophagy, differentiation, and inflammation (33,34). In terms of cell inflammatory response, NF-κB could transfer to nuclear stimulating expressions of inflammatory cytokines (35). In our study, we found that LPS single treatment significantly up-regulated the expressions of IκBα, p65, p-JNK, p-ERK, and p-p38 in RPTECs, and BAI co-treatment obviously alleviated these increases. Moreover, PMA, an activator of NF-κB (23), incubation remarkably alleviated the effects of BAI on LPS-induced inflammatory cytokine expressions. These results indicated that BAI exerted anti-inflammatory and anti-injury effects on the LPS-induced TIN cell model at least in part by inactivating NF-κB and MAPK signaling pathways.

To conclude, our research verified that BAI alleviated inflammatory injury in TIN patients and LPS-induced RPTECs by down-regulating NF-κB and MAPK pathways. This study explored the anti-inflammatory effects of BAI and provided a theoretical basis for its use in the treatment of TIN.
